# Timing of Breakfast, Lunch, and Dinner. Effects on Obesity and Metabolic Risk

**DOI:** 10.3390/nu11112624

**Published:** 2019-11-01

**Authors:** Jesus Lopez-Minguez, Purificación Gómez-Abellán, Marta Garaulet

**Affiliations:** 1 Department of Physiology, University of Murcia, 30100 Murcia; Spain; jesus.lopez5@um.es (J.L.-M.); puriki4@hotmail.com (P.G.-A.); 2 IMIB-Arrixaca, 30120 Murcia, Spain

**Keywords:** circadian rhythms, food timing, melatonin, nutrigenetic, obesity, weight loss

## Abstract

(1) Background: Eating is fundamental to survival. Animals choose when to eat depending on food availability. The timing of eating can synchronize different organs and tissues that are related to food digestion, absorption, or metabolism, such as the stomach, gut, liver, pancreas, or adipose tissue. Studies performed in experimental animal models suggest that food intake is a major external synchronizer of peripheral clocks. Therefore, the timing of eating may be decisive in fat accumulation and mobilization and affect the effectiveness of weight loss treatments. (2) Results: We will review multiple studies about the timing of the three main meals of the day, breakfast, lunch and dinner, and its potential impact on metabolism, glucose tolerance, and obesity-related factors. We will also delve into several mechanisms that may be implicated in the obesogenic effect of eating late. Conclusion: Unusual eating time can produce a disruption in the circadian system that might lead to unhealthy consequences.

## 1. Introduction

Obesity treatment has undergone numerous changes, but problems of attrition and variability in response remain. Up to the 1960s, hypocaloric diets were the only recommended treatment, while the 1970s saw the introduction of behavioral therapy (BT), promoting changes in lifestyle and eating habits as an alternative therapy [1,2]. Since then, many studies have underlined the importance of BT together with dietetic treatment in all forms of weight control. Despite the many widely attested benefits associated with weight loss, the usefulness of dietetic treatment has been questioned [3] since some studies have shown that as many as 80% of patients abandon treatments before achieving their goal [4].

Weight loss and attrition in response to behavioral–dietary interventions show a wide range of inter-individual variation [5,6]. While the type of diet [7], exercise level [8], and emotional factors [6] contribute to differences in weight-loss effectiveness, little is known about additional causal factors. We have discovered that the timing of food intake is an emerging factor that may predict the success of weight loss therapies. Not only “what” but also “when” we eat may have a significant role in obesity treatment [9].

We found that eating the main meal late (after 3 p.m.), was predictive of difficulty in weight loss [9]. In addition, the distribution of energy intake across meals may be an important factor. As Jakubowicz et al. have shown, those subjects assigned to a small breakfast and a large dinner lost significantly less weight than those assigned to a large breakfast and a small dinner [10]. Furthermore, we have shown that food timing may affect other circadian-related variables that can predict weight loss [5,11]. We have reported genetic contributions to these factors, finding that circadian-related single-nucleotide polymorphisms (SNPs) are associated with weight loss effectiveness [12,13], adherence [14], and food timing [15]. We have also observed food timing and genetic interactions of SNPs in obesity that may predict weight loss and weight loss trajectory. Together, these observations suggest that eating at the wrong time may negatively influence the success of obesity treatments and several mechanisms may be involved in the obesogenic effect of eating late.

In this review, we discuss the timing of the three main meals of the day, breakfast, lunch and dinner, and the impact that eating during the biological night can have on metabolism, glucose tolerance, and obesity-related factors.

## 2. Lunch Timing Affects Weight Loss Effectiveness

One of the first studies that have highlighted the potential impact of food timing on metabolism has been conducted by the group of Turek in 2009 [16]. In that study, those mice that were fed with a high-fat diet during the “right” feeding time (during the dark period in rodents) gained less weight than those fed with a similar high fat diet but during the “wrong” period (light period in rodents, when feeding is normally reduced). This study inspired our group together with Dr. Scheer to develop a similar observational study in humans, in order to determine whether food timing influences body weight during a dietary treatment to obesity [15].

**Figure 1:**
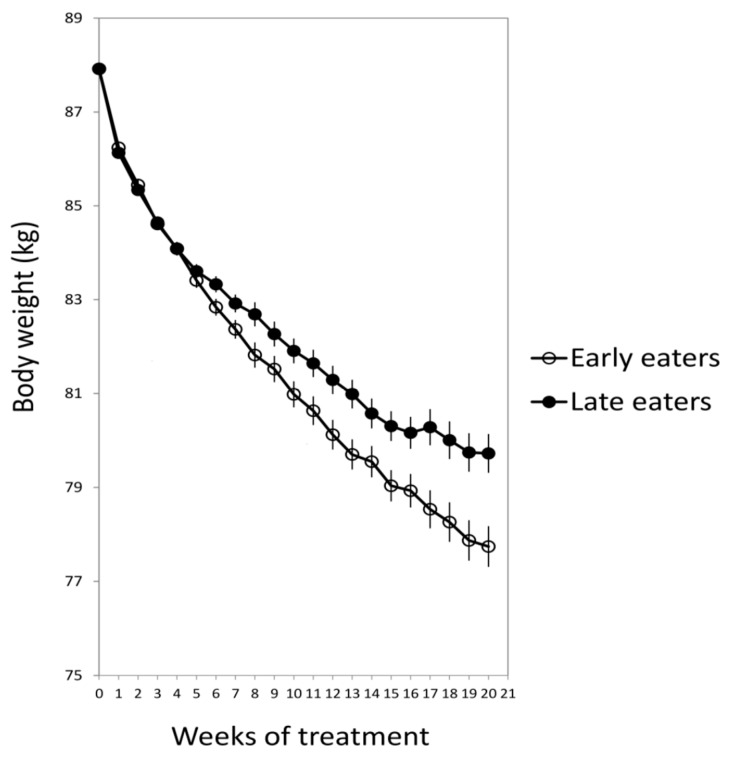
The weight loss evolution of late and early lunch eaters during the 20 weeks of treatment. Adapted from Garaulet et al., 2013 [15].

For this purpose, 420 obese subjects who attended different nutritional clinics in Spain to lose weight were classified regarding the timing of the main meal of the day (lunch in Spain). Results showed that late lunch eaters (after 3 p.m.) lost less weight during the treatment than early lunch eaters (before 3 p.m.), in spite of having similar age, appetite hormones, energy intake and expenditure, sleep duration or macronutrients distribution (Figure 1) [15]. It was remarkable that late eaters were more evening type, i.e., evening types stay up late at night, rise at a later time in the morning, and perform best mentally and physically in the late afternoon or evening [17] and carried the risk variant at *CLOCK* rs4580704 more frequently [15]. This study encouraged us to delve into the importance of meal timing on metabolism, obesity and weight loss, and opened a new door for further studies in this field that has been named “Chrononutrition” (Table 1).

One question that arose from this study was whether the effect of food timing on weight loss was only present in dietary treatments for obesity–based on low-calorie diets–or it was also present after bariatric surgery for obesity [38]. After surgery, patients may be classified in (A) “Good weight loss responders”, those who lose about 80% of their excess weight during the first year after surgery and maintain their weight loss after six years of follow-up, (B) “Secondary bad responders”, those who lose about 80% of their excess weight during the first year after surgery but regain about 40% of their initial excess weight after six years of follow-up, and (C) “Primary bad responders” those who lose only about 40% of their initial excess weight during the first year after surgery. This study lead by Izquierdo-Pulido explored several characteristics of these patients, such as energy intake, macronutrient composition, physical activity levels, appetite hormones and sleep duration [38]. Our results showed that from these lifestyle factors, the timing of food intake was the only factor that could predict weight-loss effectiveness. In agreement with our previous study performed in dietary treatment for obesity, after bariatric surgery, late lunch eaters (after 3 p.m.) lost less body weight after surgery than early eaters. Indeed, the frequency of late eaters was significantly higher among the “Primary bad responders” (70%) than among the “Good weight loss responders” or “Secondary bad responders” (~30%). Although these observational studies point to food timing as a relevant factor in the effectiveness of obesity treatment (dietary and surgery), we cannot discard the possibility that people who are predisposed to gain weight due to physical inactivity or other lifestyle or physiological factors [39] consume food at different times of the day than those who are not.

## 3. How Does the Timing of Food Intake Affect Metabolism?

In order to understand the mechanisms that underlie the difficulties of late eaters in losing weight, we developed a crossover randomized trial in 32 young women studied under two lunch-timing conditions: Early eating (lunch at 1 p.m.) and late eating (lunch at 4:30 p.m.). Volunteers received standardized meals during both meal interventions. Late eating decreased glucose tolerance, resting energy expenditure, and carbohydrate oxidation as compared to early eating. Besides, the cortisol profile was blunted for late eating as compared to early eating, similarly to that found under acute stress situations [11]. Eating late also affected the daily rhythms of peripheral temperature, towards a similar pattern to that found in overweight/obesity women which was related to metabolic alterations [40].

In order to assess whether microbiota composition and diversity were implicated in the metabolic effects of late eating, we carried out a second randomized and crossover study in 10 healthy normal-weight women [18]. We showed the impact of food timing on human salivary microbiota. There was a significant diurnal rhythm in saliva diversity across both early and late eating conditions (1 p.m. and 4 p.m., respectively) [18]. Moreover, late eating inverted the daily rhythm of salivary microbiota diversity as compared to early eating. This may have deleterious effects on the metabolism of the host [18]. It has been demonstrated that saliva bacteria, such as oral Fusobacteria, which changed with food timing, have an impact on the intestine and are related to Chron´s disease and intestinal inflammatory diseases [41]. Nevertheless, further studies should analyze the impact on obesity and weight loss of this inverted rhythm of saliva microbiota when eating late. Indeed, in spite of the significant amount of scientific studies which associate dysbiosis with obesity, even the causative role of the microbiome in obesity remains debated [42].

## 4. Timing of Food Intake Does Affect Everyone or it Depends on Genetics?

Once we knew that food timing affected BMI and weight loss efficiency, we wondered whether eating late affected everybody or by contrast the effects of meal timing on weight loss changed depending on the individual´s genetic background. In order to address this question, we selected *PERILIPIN1* (*PLIN1*), a candidate gene for obesity that encodes an adipocyte-associated protein (PLIN1) which influences body weight, fat accumulation, and lipolysis and which has been shown to display circadian rhythms in murine adipose tissue, although it has not been demonstrated in humans yet [19].

*PLIN1* promotes fat storage in adipose tissue by limiting the lipolytic activity of hormone-sensitive lipase. Our results indicated that eating late was related to less total weight loss and slower weight loss rate only in major carriers (AA) of a particular genetic variant in *PLIN1* (14995A > T) which constitute a 44% of the population who attended the nutritional clinics. Whereas food timing did not influence weight loss among T carriers. This study demonstrates that not everybody is affected in the same manner by eating late, and that genetics may play an important role in interindividual differences in weight loss depending on the timing of food intake. In the following section, we will discuss another example of the interaction between food timing, dinner timing, and genetics, which is related to one genetic variant in the melatonin receptor 1b (*MTNR1B*) [27]. These are two examples, but further studies should be performed in larger populations to discover other novel genetic variants that may interact with food timing for weight loss.

## 5. Late Dinner

Spain is one of the countries in which people have dinner the latest in Europe. Spaniards usually have dinner around 10 p.m., thus eating later than Italians (9 p.m.), Frenchmen (8 p.m.), Germans (7 p.m.) and finally Swedes, who have dinner around 6 p.m.

While our studies showed that the timing of lunch, and not the timing of breakfast or dinner, was related to weight loss effectiveness, other studies have demonstrated that having a late dinner or eating late at night associates with increased risks of obesity [20,21,22,43], dyslipidemia [21,23], hyperglycemia [24], and metabolic syndrome [22,43].

Previous research has shown that eating in misalignment with the biological clock, such as eating late at night and shift work, is associated with increased risk for diabetes [44]. Glucose metabolism shows clear diurnal variation, and small changes in meal timing, i.e., the distribution of caloric intake across the normal wake episode, appear to influence insulin resistance [11,25]. Indeed, a 12-week experimental study in overweight/obese women with metabolic syndrome randomized into two iso-caloric weight loss groups showed that subjects with the highest caloric intake during dinner had greater insulin resistance than those with the highest caloric intake during breakfast [10]. This suggested that reduced intake at dinner was beneficial and might be a useful alternative for the management of the metabolic syndrome. A large epidemiological study performed in a Japanese adult population (*n* = 61,364) of late-night-dinner eaters demonstrated that late-night-dinner eating was robustly associated with hyperglycemia independent of relevant confounders including BMI [24] (Table 1). Studies performed in laboratory conditions have found that inversion of the sleep/wake and fasting/feeding cycle, i.e., being awake and eating during their biological night, caused multiple metabolic changes including increased postprandial glucose and insulin concentrations [26,45]. In fact, postprandial responses of some of these healthy subjects during their biological night were equivalent to the responses of prediabetic individuals [45].

### 5.1. Melatonin and Late Eating

Melatonin is a hormone known for its central role in the circadian system as a signal of the biological night, and it may be involved in the detrimental effects of late eating on glucose metabolism. We have shown that melatonin administration (5 mg) worsens glucose tolerance [46]. Our results obtained in 40 overweight/obese women of European ancestry who were habitual late eaters show that those who self-reported dinner within 2.5 h of their habitual bedtime, in the presence of high endogenous melatonin levels, had a decrease in glucose tolerance. This study suggests that the concurrence of meal timing with elevated endogenous melatonin concentrations results in impaired glucose tolerance. Melatonin concentrations increase approximately half an hour before bedtime [27]. According to this, the chance of concurrence of food intake with elevated endogenous melatonin concentrations is low in those countries that have an early dinner (i.e., Sweden, Germany), while in Spain, where dinner is around 10 p.m., melatonin concentration is approximately three times higher at dinner time, particularly in young people who show higher endogenous levels than older people. This situation increases the probability of glucose-related metabolic alterations [47].

The gene that encodes the melatonin receptor 1B (*MTNR1B*) has been identified as a novel type 2 diabetes (T2D) risk gene. The common SNP at rs10830963 has been associated with one of the strongest effects on the oral disposition index–the product of insulin secretion and insulin sensitivity [48]–from the 90 common variants identified for T2D to date [49,50,51]. Our study shows that eating late impairs significantly glucose tolerance only in risk-carriers (G), which is a gain-of-function genetic variant, and not in the non-risk carriers (CC) [27]. From these studies, we concluded that eating late at night impairs glucose tolerance, mainly in those carriers of the risk allele G at *MTNR1B*, which constitute ~49% of Caucasian populations. This demonstrated that melatonin is implicated in the detrimental effects of eating late at night when melatonin levels are normally high [27,49].

### 5.2. “Circadian” Timing of Food Intake Versus “Clock” Timing

**Figure 2:**
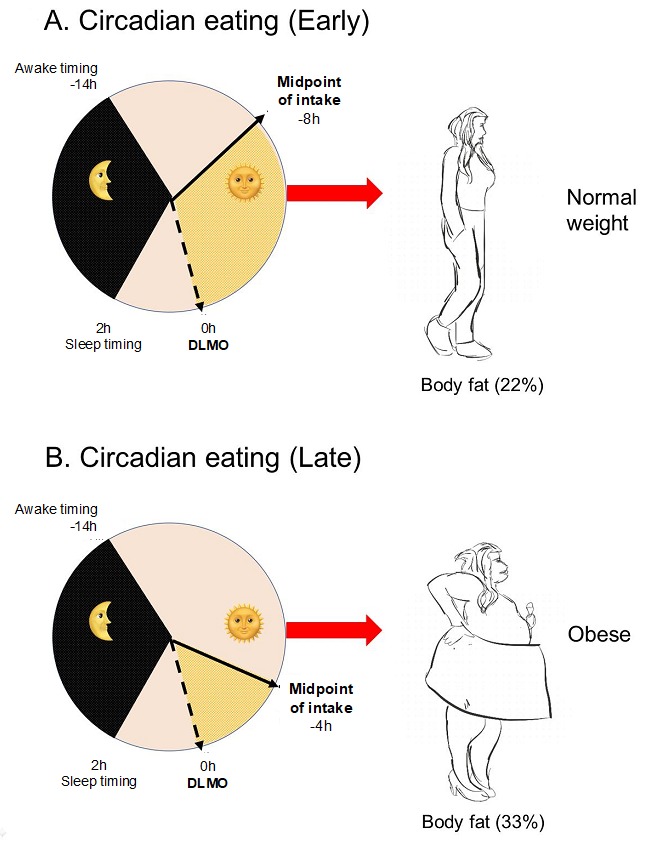
Relative timing of physiologic events in two representative subjects. (**A**) A representative participant with large phase angle (time difference) between caloric midpoint (average time at which 50% of daily calories were consumed) and Dim Light Melatonin Onset (DLMO) timing (early circadian food timing). (**B**) a representative participant with small phase angle between caloric midpoint and DLMO (late circadian food timing). The dotted line is the timing of the DLMO, the yellow shaded area denotes the phase angle, and the black shaded area denotes habitual sleep timing for that participant relative to DLMO. Adapted from Mc Hill et al., 2017 [53].

One relevant aspect of meal timing studies is to define what a late dinner is. Clock timing (external timing) may not be useful to study metabolic alterations related to dinner timing. It is known that the beginning of the biological night (internal timing), as assessed by melatonin onset under dim light conditions (Dim Light Melatonin Onset; DLMO), may differ between individuals depending on their circadian timing or chronotype [52]. For example, some subjects who are early chronotypes present early melatonin onsets (DLMO around 7 p.m.). Late chronotypes have late melatonin onsets (DLMO around 1 a.m.), while neither-types have their melatonin onset around 10 p.m. [52], with interindividual differences of six or more hours in the timing in which the biological night starts in different chronotypes. In this sense, dinner at 9 p.m. (clock time) may be a late circadian dinner for those subjects with melatonin onsets at 7 p.m., but it may be an early circadian dinner for those whose biological night starts at 1 a.m. Considering that high endogenous melatonin levels may impair glucose [27], a late dinner referred to clock time may have different metabolic effects depending on the individual´s biological night and/or the concurrence of food intake with high endogenous melatonin levels. These results were confirmed by Mc Hill AW et al. [53] who described up to 10 hours of interindividual differences in DLMO among college-aged individuals. In this study, eating late was related to BMI and body fat percentage when considering the “circadian timing” of food intake (i.e., timing of food intake relative to melatonin onset) while there were no differences between lean and non-lean individuals when considering the “clock timing” of food consumption. Moreover, students were classified based on their caloric midpoint, that is, the average time at which 50% of daily calories are consumed. Those students who had an early caloric midpoint, approximately eight hours before the melatonin onset or biological night, were lean, while those having a late caloric midpoint, approximately four hours before their biological night, were overweight/obese and had a higher body fat percentage (Figure 2).

Although DLMO is the recommended method to assess the biological night, this approach requires study participants to remain in dim light conditions for many hours and undergo repeated blood or saliva collections to measure melatonin concentrations, which is not practical for most epidemiological or clinical studies. A practical way to approximate circadian time of food intake is to consider the timing of food intake relative to the sleep/wake cycle [53]. Using this approach, we have shown that higher energy intake consumed at night (i.e., during the two hours before bedtime) increases five times the probability of being obese, especially in evening-types, while among morning-types, those who have a higher intake during the morning, two hours after waking up, have lower odds of being obese and double probability of having a healthy weight.

It is known that late chronotypes, those who tend to eat late at night, have a higher risk of metabolic disturbances, which has been related to unhealthy lifestyle factors in food intake, physical activity and sleep [54]. Indeed, evening chronotypes have more difficulties controlling the amount of food eaten, show stress while on a diet and disinhibition about some foods, mostly when they get home late, and present a greater consumption of high-fat foods and alcoholic beverages [54]. They are also engaged in less physical activity and spend longer hours sitting *per* day, and show later timings for eating, exercising, and sleeping.

One extreme example of eating during the biological night are shift workers. Shift workers have an increased risk of obesity [55] and may experience hyperphagia and a high desire for energy-dense foods [56,57]. We have demonstrated that there is an endogenous circadian rhythm in hunger and that, consistent with this circadian variation, active ghrelin levels are higher during the biological evening than during the biological morning. Furthermore, we have demonstrated that the circadian misalignment itself (12-h behavioral cycle inversion) as characterizes night shift workers, increases postprandial active ghrelin levels and appetite for energy-dense foods [58].

## 6. Breakfast

The metabolic effects of breakfast are an open question in the nutritional field depending on several aspects, such as food composition, caloric and nutritional content, and timing of intake.

### Contradictory Results in Breakfast Skipping and Weight Loss

One of the first studies on the effect of caloric distribution along the day and weight loss was the already mentioned study [10] that showed that subjects who had high caloric breakfasts (700 kcal) and low caloric dinners (300 kcal) lost significantly more weight than those who had low caloric breakfasts and high caloric dinners. In both cases, subjects followed a 12-week weight loss diet of approximately 1400 kcal, maintaining the same caloric intake for lunch and during the day. This study suggested that we should recommend a high caloric breakfast in order to lose weight [10].

In this line, it has been published that skipping breakfast is associated to unhealthy behaviors, poorer diets, and lower physical activity [59,60,61,62] and also with a higher metabolic risk, i.e., higher body mass index (BMI), larger waist circumference, higher fasting insulin and increased cholesterol and LDL levels [59,60,61,62]. This situation has also been linked to higher risk of diabetes type 2 and cardiometabolic factors independent of dietary quality [28,29,34,63,64]. One potential explanation for this deleterious effect is that skipping breakfast may be difficult to compensate later in the day, and people who do not eat the first meal of the day are reported to have higher daily intakes of fat, energy and cholesterol and lower intakes of vitamins, minerals and fiber than breakfast eaters [30]. In addition, some studies have shown a correlation with the timing of other meals [31,32,37,65].

However, in a more recent systematic review published in 2019 [35] which revises 13 trials comparing breakfast consumption with no breakfast consumption, it has been concluded that the addition of breakfast might not be a good strategy for weight loss, regardless of established breakfast habit.

Although this review must be interpreted with caution due to the relatively low quality of the included studies, the apparent contradiction among studies about the beneficial or deleterious effects of breakfast, encouraged us, together with Dr. Saxena and Dr. Dashti, to develop a Genome-Wide Association Study (GWAS) of breakfast skipping in UK Biobank (approx. 200,000 participants) and to replicate the results in other European populations (Twin UK and CHARGE) [66]. In this study, we identified six genetic variants that associated with skipping breakfast and that were implicated in caffeine, carbohydrate metabolism, and circadian clock regulation.

Using Mendelian randomization (MR), we provided evidence suggesting that genetically determined breakfast skipping was causally associated with obesity in this large population of 200,000 participants (Table 1). A limitation of observational studies is that they only allow us to look for associations between one behavior and one disease, but we cannot assess causality or directionality. For example, although studies have found an association between skipping breakfast and obesity, we cannot discard the possibility that skipping breakfast is a consequence of obesity and not a cause. Randomized controlled trials (RCT) are able to address causality, however this type of studies is difficult to perform and usually limited in statistical power, due to the rather low number of participants. Our MR findings suggest that skipping breakfast could be causal of obesity. However, results should be interpreted cautiously in light of various MR limitations including that DNA’ does not contain all the information needed to specify the phenotype [67,68,69] among others [33,70,71,72].

The timing of breakfast is another relevant aspect of metabolism since it is directly connected to fasting duration at night, which has been reported to be crucial for metabolism. Previously, our research group had demonstrated in a twin study that breakfast timing has a high heritability (56%), lunch timing presents a lower heritability (38%), and that dinner timing is not driven by genetics (0%) but determined by environmental factors [36].

Unlike lunch and dinner, which are recommended early in the day, it has been proved that to have breakfast too early may be deleterious due to melatonin levels, which may still be high in the early morning. This could be particularly problematic in *MTNR1B* subjects, carriers of the common *MTNR1B* T2D risk variant G, not only because melatonin signaling is higher due to an increased receptor expression, but also because in these subjects, the duration of elevated melatonin levels may be extended with a delayed decline in the morning [73]. This effect increases the probability of concurrence with food intake in the morning, which therefore increases also the metabolic risk, as previously discussed.

## 7. Summary

**Figure 3:**
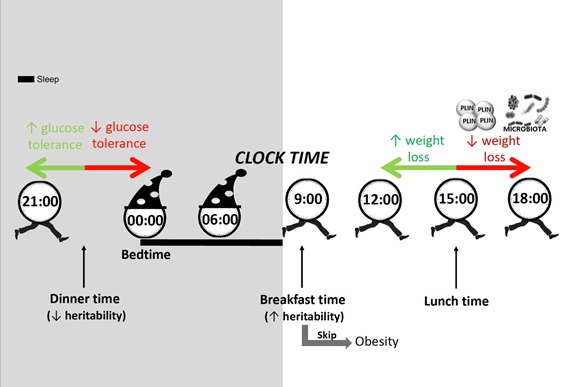
Summary of the current review. In humans, breakfast skipping is causally linked to obesity and late lunch (after 15:00 h) hinders weight loss, mainly in those carriers of a genetic variant in Perilipin (**red arrow to the right**). Late lunch eating has a deleterious effect on microbiota diversity and composition (**red arrow to the right**). Late dinner (within two hours before bedtime) decreases glucose tolerance (**red arrow to the left**). Finally, we have described some heritability studies in twins which show that dinner timing is more cultural (0% heritability), and easier to change than breakfast timing which is highly heritable (56%).

Figure 3 represents a summary of the current review. As it is shown, the timing of food intake is an external synchronizer and plays a crucial role in obesity and weight loss treatment. In humans, breakfast skipping is causally linked to obesity (GWAs in 200,000 participants) and late lunch (after 3 p.m.) hinders weight loss, mainly in those carriers of a genetic variant in Perilipin. Late lunch has a deleterious effect on microbiota diversity and composition. Late dinner (within two hours before bedtime) decreases glucose tolerance specially in G carriers of the risk allele at MTNR1B rs10830963. In addition, we have shown that individual chronotype is important in obesity. In evening chronotypes who eat at night (during the two hours before sleep) the probability of being obese increases five times, while in morning chronotypes with high caloric intake during morning hours (two hours after wake time) the probability of being obese decreases by 50%. In the current review, we have also discussed the metabolic impact of late eating when considering the endogenous “circadian timing”, relative to the beginning of the biological night or dim light melatonin onset (DLMO), or when considering the exogenous “clock time”, that may differ in specific chronotypes. Finally, we have described some heritability studies in twins which show that the timing of breakfast is hereditable while the timing of dinner is more cultural, and easier to change, whereas being an evening or morning type is mainly driven by our genetics. Nevertheless, the metabolic risk that characterizes evening chronotypes is not genetic but derives from unhealthy behaviors. Therefore, changes in these behaviors may decrease the metabolic risk in evening type subjects.

While this review considers the potential effect of food timing on obesity and weight control, most of the studies are association studies and cannot address causality. Moreover, one limitation is that dietary data from observational (e.g., longitudinal) studies are based on memory-based methods, that may fail in the assessments [74]. Further, randomized cross-over intervention studies giving a fixed diet and changing the timing of food intake should be performed to address causality.

After considering these limitations, based on the numerous epidemiological studies performed in big populations, randomized cross-over interventional studies or detailed prospective studies based in weight loss treatments followed for rather long periods of time (at least 21 weeks), our proposal is to modify the time when we eat as a potential tool to decrease obesity and metabolic risk.

## Figures and Tables

**Table 1:** Summary of the main meal timing references.

Reference	Study Type	Population	Age (Years)	Sex	Meal Timing	Metabolic Effect	Main Results	Reference
Garaulet M et al., 2013	Observational study	420 obese subjects	42 ± 11	50% Women	Lunch timing (early eaters (lunch before 3 p.m.), late eaters (lunch after 3 p.m.))	Weight loss effectiveness	1. Late eaters lost less weight and displayed a slower weight loss rate than early eaters	[15]
							2. Late eaters were more evening types, had less energetic breakfasts and skipped breakfast more frequently than early eaters	
Ruiz-Lozano T et al., 2016	Observational prospective study	270 subjects	52 ± 11	78% Women	Lunch timing (early eaters (lunch before 3 p.m.), late lunch eaters (lunch after 3 p.m.))	Weight loss evolution after bariatric surgery	1. The percentage of late eaters was significantly higher in the primarily poor weight-loss-responders (~70%) than in both secondarily poor weight-loss-responders (~42%) and good weight-loss-responders (~37%)	[17]
							2. Primarily poor weight-loss-responders had lunch later as compared to good and secondarily poor weight-loss-responders	
Bandín C et al., 2015	Randomized, crossover trial	32 subjects	24 ± 4	Women	Lunch timing (early eaters (lunch at 1 p.m.), late eaters (lunch at 4:30 p.m.))	Energy-expenditure, glucose-tolerance and circadian-related variables	1. Eating late is associated with decreased resting-energy-expenditure, decreased fasting carbohydrate oxidation, decreased glucose-tolerance, blunted daily profile in free cortisol concentrations, and decreased the thermal effect of food on wrist temperature	[11]
Collado M.C et al., 2018	Randomized, crossover trial	10 subjects	25 ± 6	Women	Lunch timing (early eaters (lunch at 2 p.m.), late eaters (lunch at 4 p.m.))	Daily rhythms of human salivary microbiota	1. Eating the main meal late inverts the daily rhythm of salivary microbiota diversity which may have a deleterious effect on the metabolism of the host	[18]
Garaulet M et al., 2016	Observational study	1287 subjects	39 ± 12	82% Women	Lunch timing (12 p.m. until 4:30 p.m.)	Weight-loss effectiveness	1. Variability at the *PLIN1* locus is associated with variability in weight loss	[19]
							2. Eating late is related to lower weight-loss effectiveness among carriers of the AA genotype at the *PLIN1* 14995AT variant	
Xiao Q et al., 2019	Observational study	872 subjects	≥18	53% Women	24-h dietary recalls (during one year every two months)	Interaction with macronutrient intake and chronotype	1. Higher dietary consumption after waking up and lower consumption close to bedtime associate with lower BMI, but the relationship differs by chronotype 2. A higher percentage of carbohydrates and protein close to bedtime was associated with higher odds of being overweight or obese, particularly in people with a later chronotype	[20]
Yoshida J et al., 2018	Longitudinal study	8153 subjects	47 ± 8	60% Men	Night eating (“dinner before bed” (within two hours before bedtime) and “snacks after dinner” (snacks after dinner)	Night eating habits and metabolic syndrome	1. In women, there was an association between eating habits at night and metabolic syndrome	[21]
							2. Night eating habits were associated with dyslipidemia in men and women	
Kutsuma A et al., 2014	Observational study (Cross-sectional)	60,800 subjects	41 ± 12	67% Men	Breakfast (skipping) and late-night eating (within two hours of bedtime)	Breakfast skipping, late-night-dinner eating, and metabolic syndrome	1. Skipping breakfast alone and late-night-dinner alone were not associated with metabolic syndrome	[22]
							2. Habitual breakfast skipping concomitant with late-night eating may represent poorer eating behavior than skipping breakfast alone and associated with metabolic syndrome	
Chen HJ et al., 2019	Observational study (Cross-sectional)	1283 subjects	≥19	56% Men	Energy intake at different times (morning (5–9 a.m.), noon (11:30 a.m.–1:30 p.m.), evening (5:30–8:30 p.m.))	Total and LDL cholesterol levels	1. Transferring 100 kcal of energy or fat intake at night to the morning or noon decreased LDL cholesterol 2. Elevated total and LDL cholesterol were positively associated with nighttime energy and fat intake	[23]
Nakajima K et al., 2015	Observational study (Cross-sectional)	61,364 subjects	46 ± 10	66% Men	Breakfast (skipping) and late-night eating (within two hours of bedtime)	Night eating, skipping breakfast and hyperglycemia	1. Hyperglycemia in the general Japanese population associated with late-night dinner eating alone, but not with breakfast skipping alone	[24]
Morgan L M et al., 2012	Randomized crossover study	6 subjects	30 ± 4	67% Women	Energy intake at different times (low glycemic index and high glycemic index, morning and night, at breakfast (9:30 a.m.), lunch (1:30 p.m.), dinner (8:30 p.m.))	Meal timing and glycemic index on glucose and insulin secretion	1. Lower insulin sensitivity in high energy consumed in the evening	[25]
							2. Both meal timing and glycemic index affected glucose tolerance and insulin secretion	
Jakubowicz et al., 2013	Randomized, open-label, parallel-arm study	93 subjects	46 ± 7	Women	Energy intake at different times (breakfast (8 a.m.), lunch (1 p.m.), dinner (7 p.m.))	High caloric intake at breakfast vs. dinner influences weight loss	1. High-calorie breakfast with reduced intake at dinner is beneficial	[10]
Rubio-Sastre P et al., 2014	Placebo-controlled, single-blind design study	21 subjects	24 ± 6	Women	Morning (9 a.m.) and night melatonin supplementation (9 p.m.)	Melatonin administration impairs glucose tolerance	1. Acute melatonin supplementation (5 mg) impaired glucose tolerance in both, morning and evening time	[26]
Lopez-Minguez J et al., 2017	Randomized, crossover trial	40 subjects	42 ± 10	Women	Dinner timing (early dinner (8 p.m.), late dinner (11 p.m.))	Late dinner and glucose tolerance	1. The concurrence of meal timing (late dinner) with elevated endogenous melatonin concentrations impaired glucose tolerance	[27]
							2. The effect was stronger in *MTNR1B* risk-carriers (GG) than in non-carriers (CC)	
Smith KJ et al., 2010	Longitudinal study	2184 subjects	7 to 15 26 to 36	53% Women	Skipping breakfast	Cardiometabolic risk factors	1. Those who skipped breakfast in both childhood and adulthood had higher waist circumference and higher fasting insulin, total cholesterol, and LDL cholesterol concentrations than did those who ate breakfast	[28]
							2. Skipping breakfast over a long period may have detrimental effects on cardiometabolic health	
Reutrakul S et al., 2014	Observational study	194 subjects	54 ± 13	71% Women	Skipping breakfast	Chronotype and glycemic control in type 2 diabetes	1. Breakfast skipping is associated with a later chronotype	[29]
							2. Later chronotype and breakfast skipping both contribute to poorer glycemic control, as indicated by higher glycosylated hemoglobin (HbA1C) levels	
Jakubowicz et al., 2012	Randomized crossover study	193 subjects	47 ± 7	60% Women	Energy intake timing (low carbohydrate diet, low carbohydrate breakfast, and high carbohydrate enriched breakfast diet)	Weight loss, ghrelin levels, and appetite scores	1. A high carbohydrate and high protein breakfast may prevent weight regain by reducing diet-induced compensatory changes in hunger, cravings and ghrelin suppression	[30]
de Castro JM et al., 2004	Observational study	886 subjects	36 ± 14	57% Women	Energy intake at different times (6–9:59 a.m., 10 a.m.–1:59 p.m., 2–5:59 p.m., 6–9:59 p.m., 10:00 p.m.–1:59 a.m.)	Food intake influences overall intake	1. Energy intake in the morning is particularly satiating and can reduce the total amount ingested for the day	[31]
							2. Energy intake in the late-night lacks satiating value and can result in greater overall daily intake	
Kant AK et al., 2015	Observational study	13,298 subjects	≥20	52% Men	Skipping breakfast (energy intake at different times)	Eating behaviors, time of eating, and dietary intake	1. Lunch meal provided more energy on the no-breakfast day than on the breakfast day	[32]
Lopez-Minguez J et al., 2019	Observational study	106 subjects	52 ± 6	Women	Timing of food intake	Heritability of the timing of food intake	1. Genetic factors contributed to a higher degree to the timing of breakfast (56%) than the timing of lunch (38%) or dinner (0%)	[33]
Mekary RA et al., 2013	Observational study	1560 subjects	66 ± 7	Women	Skipping breakfast (energy intake at different times)	Eating patterns and type 2 diabetes risk	1. Irregular breakfast consumption was associated with a higher type 2 diabetes risk	[34]
Dashti HS et al., 2019	Observational study	193,860 subjects	≥19	55% Women	Skipping breakfast	Genetic variants of skipping breakfast	1. Proxy-phenotype Genome-Wide Association Study (GWAS) identified six genetic variants for breakfast skipping, linking clock regulation with food timing	[35]
							2. Skipping breakfast was causal of obesity	
Lane JM et al., 2016	Observational study	100,420 subjects	40–69 years	55% Women	Chronotype	Genetic variants of chronotype	1. The study reports the discovery of 12 genetic loci associated with chronotype	[36]
Sievert K et al., 2019	Systematic review and meta-analysis	12 studies	≥18	70% Women	Regular breakfast consumption	Weight change and energy intake	1. The addition of breakfast might not be a good strategy for weight loss	[37]
							2. Caution is needed when recommending breakfast for weight loss in adults, as it could have the opposite effect	
